# Desmoplakin-Related Arrhythmogenic Cardiomyopathy Carriers and Desmosomal-Type Acantholysis: A Previous Description of the Keratinocyte “Fingerprint Sign”. Comment on Metze et al. Desmosomal-Type Acantholysis—A New Histologic Pattern Related to Mutations of Genes for Desmosomal Proteins. *Dermatopathology* 2026, *13*, 17

**DOI:** 10.3390/dermatopathology13030039

**Published:** 2026-08-31

**Authors:** Ángel Fernández-Flores

**Affiliations:** 1 Department of Anatomic Pathology, Hospital Universitario El Bierzo, 24404 Ponferrada, Spain; dermatopathonline@gmail.com; 2 Department of Anatomic Pathology, Hospital de La Reina, 24400 Ponferrada, Spain

We read with great interest the article by Metze et al., “Desmosomal-Type Acantholysis—A New Histologic Pattern Related to Mutations of Genes for Desmosomal Proteins”, recently published in *Dermatopathology* [1]. The authors should be congratulated for delineating and systematizing a distinctive histopathologic pattern associated with inherited disorders of desmosomal proteins. Their concept of “desmosomal-type acantholysis” is highly useful, since it separates this pattern from conventional pemphigus-like acantholysis, spongiotic dermatitis, acantholytic dyskeratosis, and epidermolytic disorders.

We would like, however, to draw attention to a previously published study from our group that is closely related to this concept. In 2021, we reported a cohort of heterozygous carriers of truncating desmoplakin (DSP) variants associated with arrhythmogenic cardiomyopathy [2]. Although these patients had no relevant macroscopic abnormalities of skin or hair, they showed a reproducible subclinical cutaneous phenotype. Histopathologic examination demonstrated widening of intercellular spaces, loss of intercellular adhesion, and focal acantholysis in the spinous layer. Immunohistochemistry showed absent or markedly reduced desmoplakin expression in most samples. In addition, trichogram analysis disclosed pseudomonilethrix in several patients.

A particularly distinctive finding in our study was an unusual cytoplasmic change in keratinocytes, consisting of a striated cytoplasmic pattern reminiscent of dermatoglyphic lines. We proposed the term “fingerprint sign” for this feature. This cytoplasmic alteration was present in all evaluable cases and was not accompanied by papillomatosis, dyskeratosis, transition cells, pale cytoplasm, or eosinophilic cytoplasmic globules. Ultrastructural analysis further supported a disturbance of keratinocyte adhesion, showing enlargement of intercellular spaces, elongation of cytoplasmic bridges, and a reduced number of desmosomes in a subset of patients.

In this regard, our findings appear to represent an earlier description of a DSP-related histopathologic pattern that overlaps substantially with what Metze et al. now define as desmosomal-type acantholysis. The features described in our cohort correspond particularly well to their category, characterized by widening of intercellular spaces in the stratum spinosum, partial loss of intercellular bridges, preserved keratinocyte differentiation, and absence of true spongiotic pallor or serous exudation. However, the cohort we studied differs from many of the syndromic examples emphasized by Metze et al., because our patients were heterozygous DSP truncating variant carriers with arrhythmogenic cardiomyopathy and a largely subclinical cutaneous phenotype.

Metze et al. do mention that heterozygous carriers may show isolated arrhythmogenic cardiomyopathy without cutaneous manifestations [1]. Nevertheless, our previous observations suggest that, at least in some such patients, the skin may show subtle but reproducible microscopic abnormalities even in the absence of clinically apparent skin disease. Therefore, heterozygous DSP-related arrhythmogenic cardiomyopathy carriers may deserve explicit inclusion within the expanding spectrum of desmosomal-type acantholysis, particularly as a subclinical or minimally expressed cutaneous phenotype.

We believe that integration of our previously described “fingerprint sign” into the broader framework proposed by Metze et al. could further refine the histopathologic spectrum of desmosomal disorders. This may be relevant not only for dermatopathology but also for the multidisciplinary evaluation of patients with desmoplakin-related cardiomyopathy, especially when cardiac findings are incomplete, genetic variants are difficult to interpret, or extracardiac markers may assist clinical assessment.

In conclusion, we fully support the concept of desmosomal-type acantholysis proposed by Metze et al. and suggest that the subclinical cutaneous phenotype of heterozygous DSP truncating variant carriers, including the keratinocyte “fingerprint sign”, should be considered part of this spectrum. Recognition of this prior observation may help complete the nosologic and morphologic framework of desmosomal-type acantholysis.

## Data Availability

No new data were created or analyzed in this study. Data sharing is not applicable to this article.
